# Investigation of Mechanical and Physical Properties of Big Sheep Horn as an Alternative Biomaterial for Structural Applications

**DOI:** 10.3390/ma14144039

**Published:** 2021-07-20

**Authors:** Tajammul Hussain M. Mysore, Arun Y. Patil, G. U. Raju, N. R. Banapurmath, Prabhakar M. Bhovi, Asif Afzal, Sagr Alamri, C Ahamed Saleel

**Affiliations:** 1 School of Mechanical Engineering, KLE Technological University, Hubballi 580031, Karnataka, India; tajammulhusain67@gmail.com (T.H.M.M.); raju_gu@kletech.ac.in (G.U.R.); nrbanapurmath@gmail.com (N.R.B.); prabhakar_mb@kletech.ac.in (P.M.B.); 2 B.V.B. College of Engineering and Technology, Mechanical Engineering, KLE Technological University, Hubballi 580031, Karnataka, India; 3 Department of Mechanical Engineering, P. A. College of Engineering, Affiliated to Visvesvaraya Technological University, Belagavi 574153, Mangaluru, India; 4 Department of Mechanical Engineering, College of Engineering, King Khalid University, P.O. Box 394, Abha 61421, Saudi Arabia; salamri@kku.edu.sa (S.A.); ahamedsaleel@gmail.com (C.A.S.); 5 Department of Mechanical Engineering, The University of Akron, Akron, OH 44325, USA

**Keywords:** big sheep horn, Deccani, mechanical property, physical property, simulation, structural application

## Abstract

This paper investigates the physical and mechanical properties of bighorns of Deccani breed sheep native from Karnataka, India. The exhaustive work comprises two cases. First, rehydrated (wet) and ambient (dry) conditions, and second, the horn coupons were selected for longitudinal and lateral (transverse) directions. More than seventy-two samples were subjected to a test for physical and mechanical property extraction. Further, twenty-four samples were subjected to physical property testing, which included density and moisture absorption tests. At the same time, mechanical testing included analysis of the stress state dependence with the horn keratin tested under tension, compression, and flexural loading. The mechanical properties include the elastic modulus, yield strength, ultimate strength, failure strain, compressive strength, flexural strength, flexural modulus, and hardness. The results showed anisotropy and depended highly on the presence of water content more than coupon orientation. Wet conditioned specimens had a significant loss in mechanical properties compared with dry specimens. The observed outcomes were shown at par with results for yield strength of 53.5 ± 6.5 MPa (which is better than its peers) and a maximum compressive stress of 557.7 ± 5 MPa (highest among peers). Young’s modulus 6.5 ± 0.5 GPa and a density equivalent to a biopolymer of 1.2 g/cc are expected to be the lightest among its peers; flexural strength 168.75 MPa, with lowest failure strain percentage of 6.5 ± 0.5 and Rockwell hardness value of 60 HRB, seem best in the class of this category. Simulation study identified a suitable application area based on impact and fatigue analysis. Overall, the exhaustive experimental work provided many opportunities to use this new material in various diversified applications in the future.

## 1. Introduction

Horns are the Bovidae family’s defensive weapons (ex. sheep, cattle, goat, buffalo, and antelope) [1,2]. In ancient history, the evolution of horn shape, size, and structure are unique for each category [3]. However, the core material composition remains common among all, i.e., keratin [4,5]. Keratin-based mineralized tissues are the lightest available material as compared to mineralized segments of bones and teeth in animals [6,7,8]. Mechanical properties such as Young’s modulus, yield strength, and impact resistance are predominant features related to horns of animals. On average, the life span of bighorn sheep is around 13 years [9], to survive among its community with territory control, fight with competitors for continuing the cycle of production, and to balance the natural activities. The impact force exerted by adult male sheep is around 3400 N and still capable of maintaining its physique. This is considered futuristic for biological safety and impacts resistance structures in crashworthiness tests for automotive and aerospace applications [10]. There are two types of keratins found in sheep horn, known as α-keratin and β-keratin [11,12]; α-keratin found in horns, hooves, hair, wool, and claws as a structural protein. However, β-keratin is much stronger and more rigid than its predecessor [5]; α-keratin is anisotropic in the radial direction (transverse direction) [13], and the keratin sheath has sulfur cross-links as a chemical bonding between matrix and fiber [4]. The protein comparison varies from the bone, tooth, and nacre, usually having mineral content for higher stiffness [14,15,16].

On the other hand, keratin recycling and utilization in an industrial application have become a recent interest in many countries. Hen feathers, a severe and unnoticed problem of poultry farms and industries that are accumulated in waste dump yards outside the cities, do not degrade quickly [17]. Yak horn sheath is another remarkable material with the best-in-class ductility and less severe plastic deformation [18]. Keratin’s moisture absorption is likely to deteriorate its strength and makes it slightly brittle to ductile [19]. Many researchers have illustrated that the increase in strain to keratin’s failure reduces strength and stiffness [20,21,22]. In earlier studies at an intermediate level of moisture gain, it resulted in better fracture toughness as compared to its dry condition; this is due to hydrogen bonding of matrix and fiber. This supports the elongation aspects of the material [21,23].

Based on earlier researchers’ study of sheep horn, breeds originating from Indian territory were not subjected to testing their capability. In India, from Karnataka state, the Haveri district breed named “Deccani” is considered for the study. It is a local breed but used from ancient days in competitive fight events; these studies were yet to explore horn mechanical properties, to the best of the authors’ knowledge. The primary objective of this work is to identify the mechanical and physical properties of “Deccani” sheep horn breeds. In the study of mechanical properties, the focus is mainly on tensile, flexural, compression, hardness, and impact behavior. On the other hand, density and moisture absorption are extracted for physical properties. Further, comparative study with statistical analysis using two-way ANOVA for tensile strength was carried out and later comparison made with various other country breeds to validate the properties. At the end, a simulation study covering fatigue and impact analysis was performed to identify the suitable application in the public domain. Overall, the work extensively compares the physical and mechanical properties with various other country breeds and makes a statement for feasible alternative material usage in industrial applications.

## 2. Materials and Methods

### 2.1. Materials

**Figure 1:**
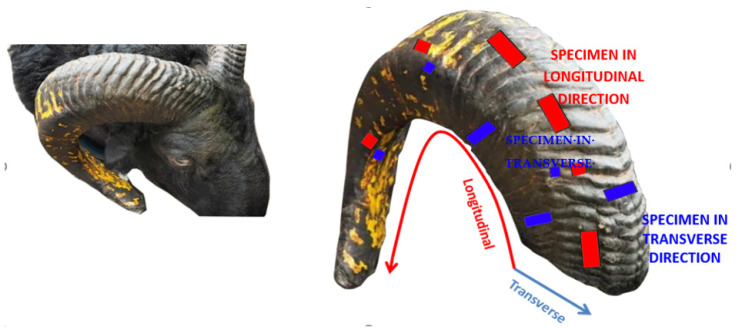
Deccani breed and slicing method for sheep horn.

**Figure 2:**
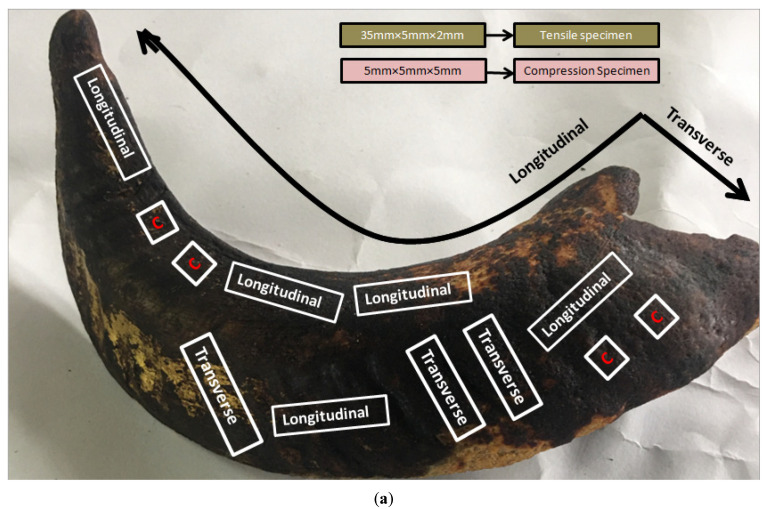

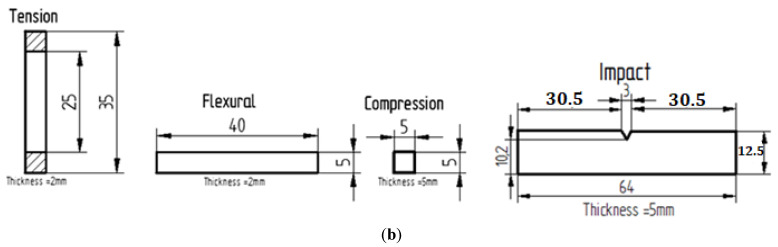
(**a**) A pattern of slicing for extraction of coupons and (**b**) ASTM standard used for slicing the horn sheath.

The two big horns of 1.5 year-old Deccani breed sheep (Karnataka sheep; *Ovis canadensis*) were extracted within 24 h after slaughter from a local slaughterhouse (used for dietary reasons; Hubbali Taluq, Dharwad District, Karnataka, India), as shown in Figure 1 (left). The horns were approximately 70 cm in longitudinal length and 18 cm in diameter at the base; the horn sheath’s thickness was irregular, as depicted in Figure 1 (right view) where the red rectangle represents the specimen in the longitudinal direction and the blue rectangle indicates the specimen in the transverse direction. The horns were cold-stored in a controlled environment before extraction of specimens from the horn sheath. The pattern of slicing for extracting the maximum number of specimens is shown in Figure 2a. The ASTM standard was used for slicing the Deccani horn sheath, as shown in Figure 2b.

### 2.2. Methodology

**Figure 3:**
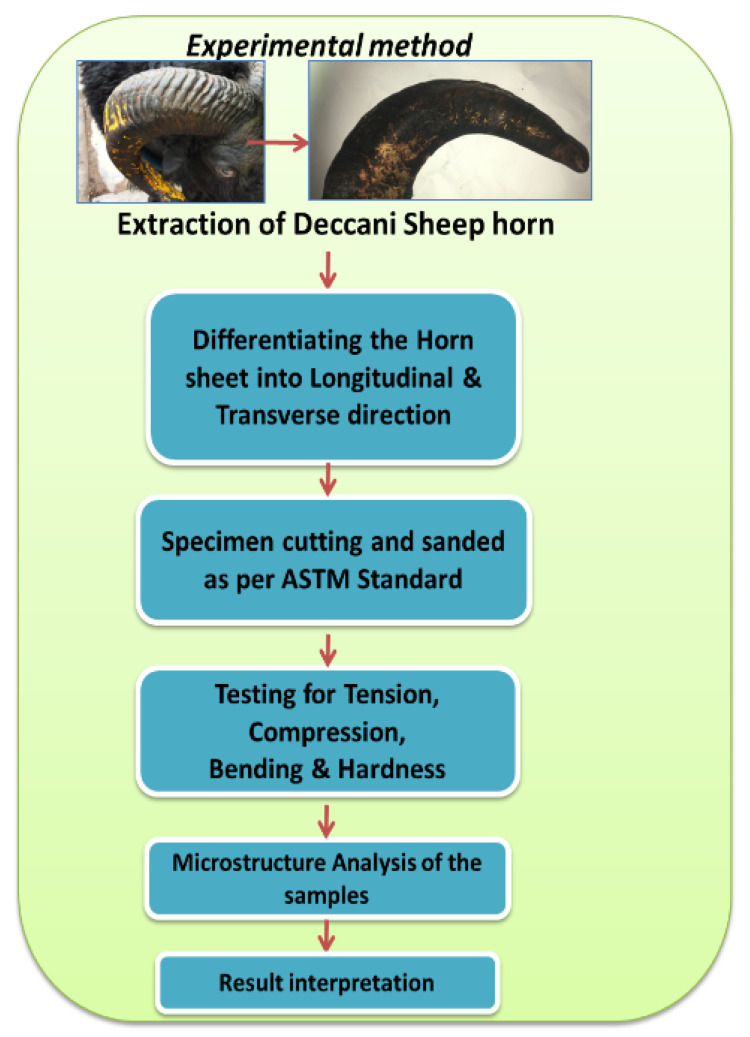
Experimental process methodology.

A roadmap for the entire research work is framed in the form of methodology. The details with the process map are shown in Figure 3. The work initiated with the extraction of a typical type of “Deccani” breed from a slaughterhouse. Each of these horns is sliced with unique optimal conditions to extract the maximum number of coupons. The specimens were extracted according to the ASTM standard and filed to achieve the exact shape and size to match the dimensions depicted in Figure 2b.

## 3. Experimental Tests

The complete experimental work comprised physical and mechanical studies. These studies are explained in the following sections.

### 3.1. Physical Testing

#### 3.1.1. Density Test

The specimen density test was performed by applying Archimedes principle using distilled water according to ASTM D792 [24]. The actual density of each sample was measured by using a cantilever setup and weighing machine.

Density is determined by using the formula

(1) $$\rho_{\mathrm{specimen}}=\frac{W_{a}}{W_{a}-W_{w}}$$

where

$\rho_{\mathrm{Specimen}}=$ Actual density of specimen (g/cc);
${\;W}_{a}=$ Weight of a specimen in the air (g);
${\;W}_{w}=\text{Weight}\;\text{of}\;a\;\text{specimen}\;\text{in}\;\text{water}\;\left(g\right)$.

#### 3.1.2. Water Absorption Test

Water absorption tests were carried out as per ASTM D5229 [25] with six coupons of specification 6 mm × 6 mm × 6 mm for four categories. Coupons were subjected to sunlight for 24 h before measuring the observations. The coupons were immersed in distilled water and weighted every 24 h of the cycle. Freshwater was utilized after daily measurement. After 11 days, the coupons were taken out of the container and dried with a cloth. The samples were then placed in a preheated oven at 110 °C for one day to dehydrate completely. During dehydration, each sample was weighed every 12 h of time-lapse.

### 3.2. Mechanical Testing

All of the specimens were extracted from the horn with a diamond saw cutting blade and sanded with 240 grit sandpaper. For each set of specimens, two samples were developed by loading in the longitudinal and transverse directions, as shown in Figure 2a. The test was carried out for each sample in ambient (dry) and fully rehydrated condition (wet) for each loading direction.

#### 3.2.1. Tensile Test

**Figure 4:**
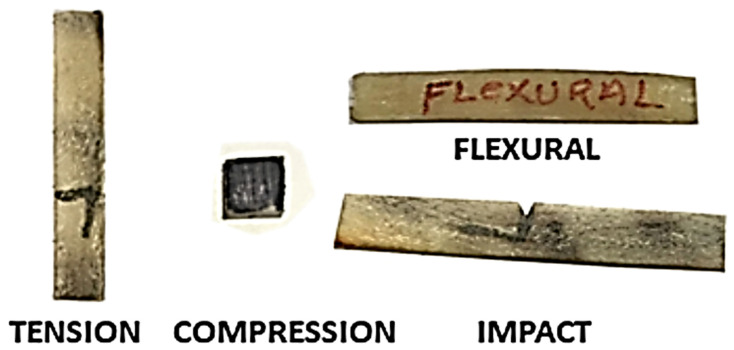
Coupons for mechanical property test.

For a total of 20 samples, 10 were longitudinally oriented and 10 transversely oriented. Among the 10, in particular, 5 were “wet” condition and the other 5 were “dry” condition [10]. The tensile test was performed using a micro universal testing machine equipped with 10 kN load cells. The miniaturized specimens were developed according to ASTM D-3039 [26]. Specimens of dimension 35 mm × 5 mm × 2 mm (length × width × thickness) were sliced from horn sheath with a diamond saw blade and sanded with 240 grit sandpaper, as shown in Figure 4. The two ends of each sample were wrapped with 100 grit sandpaper to ensure that slippage did not occur. A uniaxial load was scoped on one end of the gripper. The gauge length of 25 mm and a crosshead speed of 2 mm/min were used.

#### 3.2.2. Compression Test

Twenty cubical specimens with dimensions 5 mm × 5 mm × 5 mm were prepared for a compression test as shown in Figure 4, ten each in the longitudinal direction and the transverse direction. Five samples in the rehydrated (wet) condition and 5 in the dry condition were subjected for a test, the same accepted for the transverse specimens. The compression test was executed on the same Universal Testing Machine (UTM) where we performed the tensile test but according to ASTM: E09 [27].

#### 3.2.3. Flexural Test

**Figure 5:**
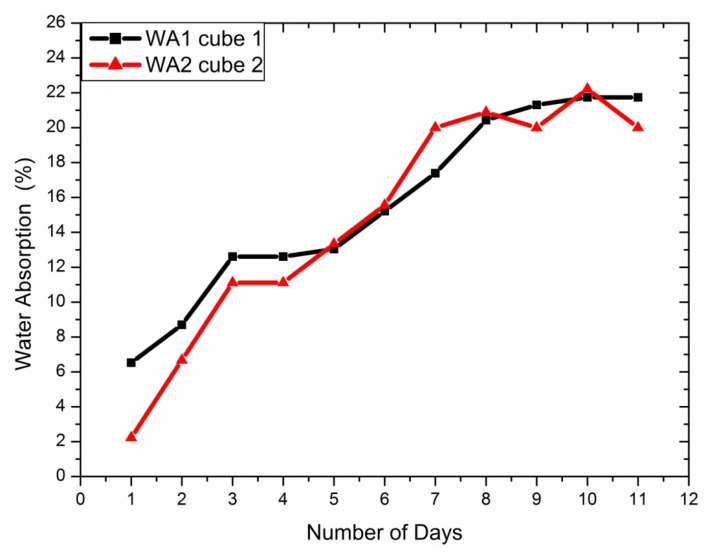
Water absorption percentage based on the number of days.

The flexural strength test provides the bending strength for a given specimen. The coupons were cut into rectangular prisms of dimensions 30 mm × 8 mm × 3 mm (length × width × thickness), as shown in Figure 5, with a diamond saw and sanded with 240 grit sandpaper. Micro Universal Testing Machine (M-UTM) manufactured by Tinius Olsen, India, was used to perform the coupon test for 10-tonne capacity following ASTM D790–07 [28]. for developing the coupons. Twenty coupons were etched to prepare two sets of samples, ten in longitudinal and ten in transverse direction. Five tests were made in the wet condition out of the ten longitudinal specimens, and the other coupons were used for the dry condition.

#### 3.2.4. Hardness Test

The hardness test was conducted with a Rockwell series tester in the material testing laboratory of KLE Technological University. ASTM D785 [29] was followed for preparing and subjecting the sample to an average of six samples per category.

#### 3.2.5. Impact Test

As per ASTM standard D256 [30], applied to plastic materials, perhaps is lower than metal-based conditions. The details about dimensions are highlighted in Figure 2b. As per the ASTM standard, method A was used while testing the specimen for the Izod case. The energy of impact can be expressed by:

(2) $$\text{Impact}\;\text{strength}\;=\frac{\text{Energy}\;\text{Absorbed}\;\left(J\right)}{\text{cross}\;\text{section}\;\text{of}\;\text{specimen}\;\text{at}\;\text{the}\;\text{Notch}\left(m^{2}\right)}$$

## 4. Results and Discussion

### 4.1. Physical Test

#### 4.1.1. Density Test

The density of the Bighorn sheep horn was measured using Archimedes principle and shown in Table 1. The weight of the specimen in air and water were determined using Equation (1) (the equation for the density in the experimental section).

#### 4.1.2. Water Absorption Test

Water absorption is a critical performance test to measure the degradation or deterioration in the sample. Table 1 shows that, on average, 20.87 is the percentage of water absorption. The goat’s new horns showed a percentage of water absorption in the range 10–12% [31,32,33,34].

However, fully hydrated big sheep horn showed 27% absorption, 38% for pronghorn, and domestic horn 21% [35]. In comparison to all the cases, the water absorption percentage is the lowest among all categories. The tests were reported for 11 days. The results are shown in Figure 5.

### 4.2. Mechanical Tests

#### 4.2.1. Tensile Strength Test

**Figure 6:**
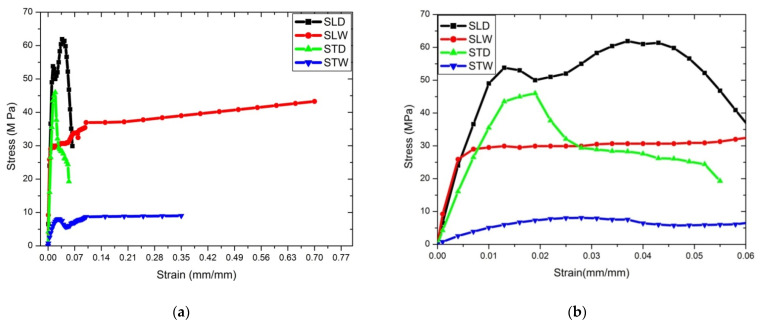
(**a**) Stress–strain curve for all samples and (**b**) stress–strain curve for closure image.

A tensile strength test was conducted for four cases; the observed details are shown in Figure 6a. The tensile yield strength observed for the longitudinal dry condition is 60 MPa, which is greater than high-density poly ethylene, ABS, polypropylene, and similar poly-methyl-methacrylate (PMMA), and higher than other fiber-reinforced polymer composites [36]. Young’s modulus of stress in the longitudinal direction of the dry specimen (SLD) is lower than nettle [37], flax [38], ramie [39], and pineapple [40], and almost equivalent to banana [41] and sisal fibre [42]. However, it is higher than cotton [43], kenaf [44], root [45], elephant grass [46]. The comparative study of various breeds of sheep horn around the world is discussed in Section 5. The details of the nomenclature of specimens used for mechanical property testing are given in Table 2.

**Figure 7:**
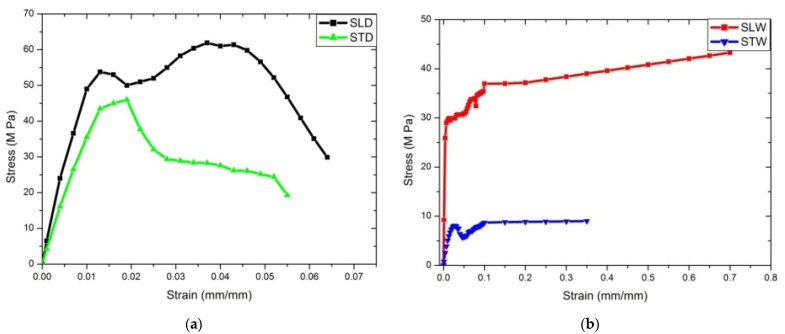
(**a**) Longitudinal and transverse stress–strain curve for dry conditions and (**b**) longitudinal and transverse stress–strain curve for wet conditions.

Figure 6b represents the closure view of the stress–strain diagram. Figure 7a represents the comparison of stress–strain diagram for the dry condition for both STD and SLD; it was observed that SLD has greater plastic deformation as compared to STD. Figure 7a represents a comparison of dry conditions alone, which shows that the yield strength and ultimate strength of the longitudinal direction is 19.2% and 26.4% more than the transverse direction. Yield and ultimate strength for STD, however, are nearly the same. From Figure 7a, it is seen that once the ultimate stress is reached in SLD and STD, the failure stress is reached faster in SLD as compared to STD. Figure 7b represents the comparison of the stress–strain diagram for the wet condition for both STW and SLW. From Figure 7b, it is seen that failure strain and toughness of SLW is maximum among all other peers. It is observed that the yield strength and ultimate strength of the longitudinal direction is 80% more than the transverse direction. In addition, the percentage of failure strain of SLW is twice that of STW. Figure 7b indicates that in the wet condition, SLW has greater strength and load-carrying capacity as compared to STW. From Figure 7a,b, it is observed that the longitudinal direction possessed greater strength in the dry condition, but more ductility in the wet condition as compared to the transverse direction. Overall, Young’s modulus for SLD is 6.5 ± 0.5 GPa, whereas it is 1.7 ± 0.5 for big sheep horn, 1.8 ± 0.2 for pronghorn, and 2.1 ± 0.6 for domestic horn [47]. The failure strain rate is 6.5% for SLD, whereas it is 14.5% is for mountain goats and 5.7% for domestic sheep, as recorded for peer cases [48,49]. However, failure strain for SLW and STW is 70 ± 0.5%, and 35 ± 0.1%, respectively, which is greater than that recorded for big sheep horn of the USA and China [50]. Comparative studies for all four cases of Deccani sheep horns are tabulated in Table 3.

However, to verify the significance of the direction (longitudinal and transverse) and condition (dry and wet) of the specimen on the mechanical properties, a two-way ANOVA analysis was implemented on the results reported in Table 3 by using Statistics Kingdom two-way ANOVA calculator. During two-way ANOVA, the effect of direction of the specimen, the effect of the condition of the specimen, and the effect of interaction between direction and condition of the specimen on yield stress, Young’s modulus, ultimate strength, and failure strain were examined separately. In Table 4 and Table 5, DF indicates the degree of freedom, SS shows the sum of squares, and MS indicates mean square. Moreover, F-value and *p*-value are important parameters to decide whether direction and condition have significant mechanical properties; based on the value of F and *p* we accept or reject the null hypothesis. If F-value, which is tabulated in Table 4 and Table 5, is greater than F-value determined by using F-table [51,52], then the null hypothesis is rejected. Second, the *p*-value is used to decide the validity of the null hypothesis. If the *p*-value is less than the level of significance, i.e., *p* < 0.05, it can be said that factors of condition and direction have a significant effect on the mechanical property. For instance, here we will study the effect of direction on yield strength, after executing two-way ANOVA, the degree of freedom in the numerator (direction) is “1” as shown in Table 4, whereas the degree of freedom in the denominator was found to be 16 by using an F-table critical value of F determined as 4.49. The F-value in Table 4, “149.9076” is greater than the critical value (=4.48), hence it leads to the rejection of the null hypothesis. After implementing the same procedure on condition as well as interaction, the significance of the condition and interaction on the mechanical properties was reported. From Table 4 and Table 5, it is concluded that the significance of the condition, direction, and interaction between them do exist on the mechanical properties of the Deccani sheep horn.

#### 4.2.2. Flexural Strength Test

**Figure 8:**
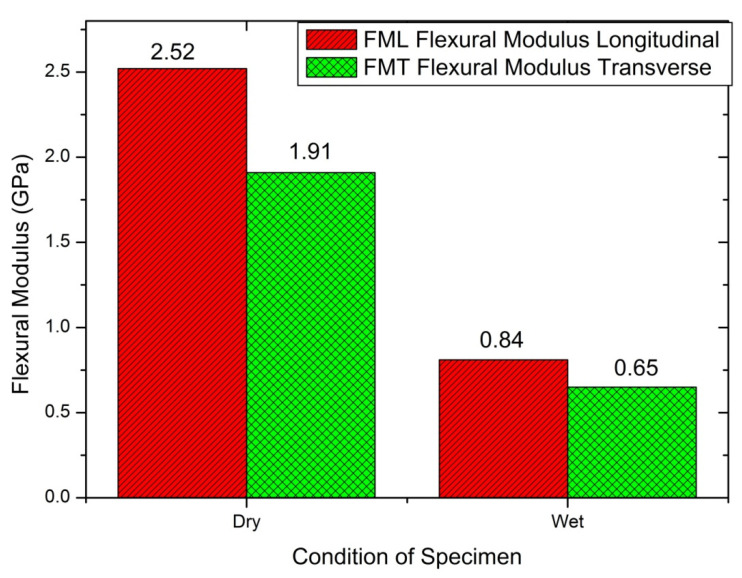
Flexural modulus determined by the three-point method for dry and wet condition in the longitudinal and transverse directions.

**Figure 9:**
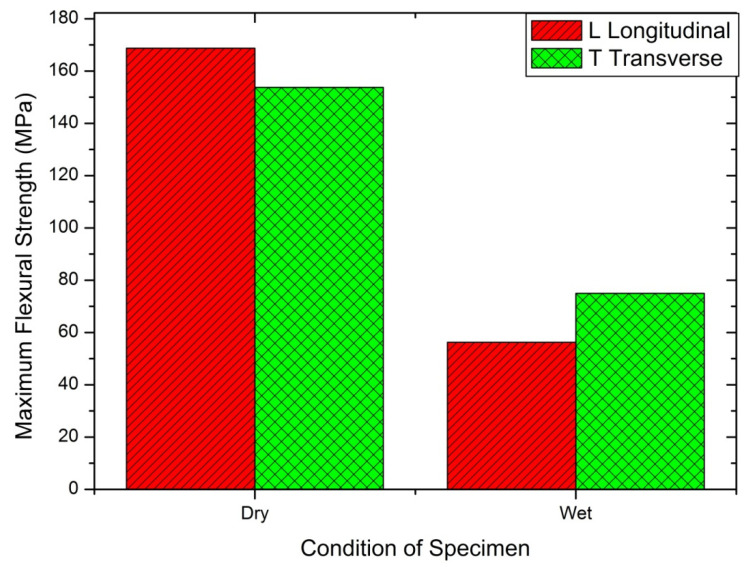
Maximum flexural strength determined by the three-point method for dry and wet condition in the longitudinal and transverse directions.

Three-point bend test conducted for dry/wet conditions was based on longitudinal/transverse cases. SLD resulted in 2.52 GPa flexural modulus for 20.8% water absorption, compared to Warburton’s [53] test result, 1.5 GPa for 20% water absorption, and Kitchener and Vincent [54] finding of 1.8 GPa for 40% water absorption. Table 6 shows the flexural properties of Deccani sheep horn. The wet sample showed almost one-third of the result with a dry sample; eventually, the brittle transitioned to ductile at the fracture period. Figure 8 and Figure 9 show the details for wet and dry conditions, and the longitudinal and transverse conditions of coupons.

The flexural strength calculation is given by Equation (3):

(3) $$\sigma =\frac{3PL}{2bd^{2}}$$

**Calculation:** Steps to calculate bending strength are given below.
Formula $\sigma =\frac{3PL}{2bd^{2}}$

Specimen Dimension40 mm×5 mm×2 mm Where Total length = 40 mm Gauge length(L) = 25 mm Width of specimen (b) = 5 mm Thickness of specimen(d) = 2 mmSample Calculation$\sigma =\frac{3PL}{2bd^{2}}=\frac{3\;\times \;0.09\;\times \;1000\;\times \;25}{2\;\times \;5\;\times \;2^{2}}=168.75\;MPa$Flexural Modulus$E_{flexural}=\frac{PL^{3}}{48I\delta }$
where; *P* = Ultimate load (N), *L* = Gauge length (mm), *b* = Width of Specimen(mm), *d* = Depth or thickness of Specimen(mm).

Table 7 represents the two-way ANOVA test results for flexural properties of Deccani sheep horns. When we compare all the F-values with critical values from the table it is clear that there is a significant effect of direction and condition on the flexural modulus of the Deccani sheep horn. Moreover, when we compare F-values and *p*-values for flexural strength it is observed that the condition, direction, and interaction of the specimen have a significant effect on flexural strength.

#### 4.2.3. Compression Test

**Figure 10:**
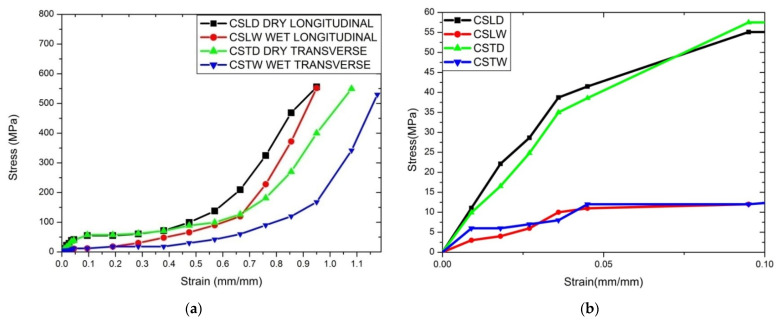
(**a**) Compression stress–strain curve for all the specimens and (**b**) compression stress–strain curve for the closure image.

**Figure 11:**
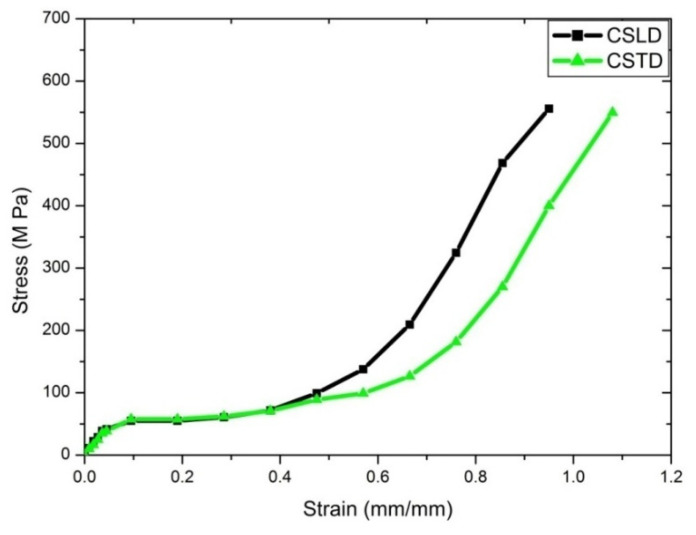
Compression stress–strain curve for dry condition.

**Figure 12:**
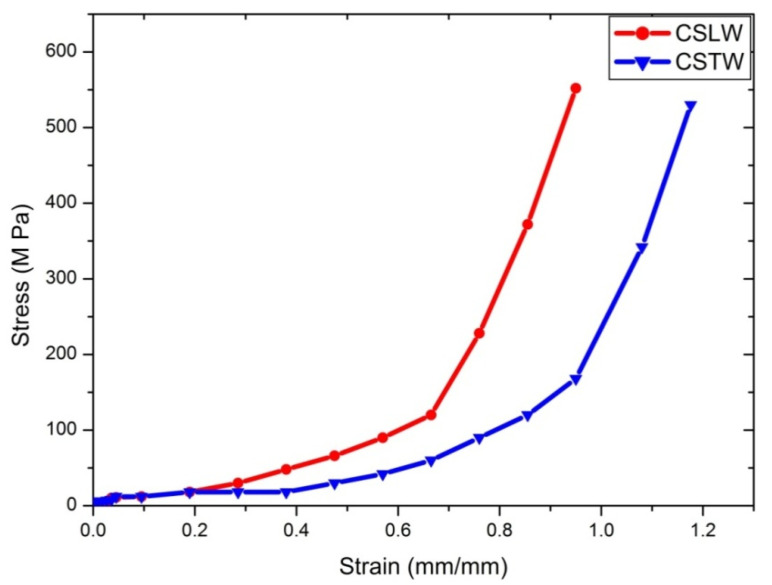
Compression stress–strain curve for wet condition.

Compression tests for each direction (longitudinal and transverse) for dry and wet conditions were done with more than six samples for each condition. Nomenclature for coupons for compressive testing is shown in Table 8. The stress–strain curves for all of the cases are depicted in Figure 10, Figure 11 and Figure 12. CSLD showed 43.5 MPa; however, 55.8 MPa [10] with Young’s modulus of 1.45 GPa was found for SLD in the case of 2.4 GPa [47] and 2.3 GPa [48]. Figure 10b represents a closure image of the compression stress–strain curve; it is observed that yield strength for CSLD was 7.32% higher than CSLD. Figure 11 represents the stress–strain curve for dry condition where it is observed that, from strain of 0.1% to 0.4%, CSLD and CSTD follow the same trend, but after 0.4% strain, CSTD exhibited more plastic deformation as compared to CSLD. Figure 12 represents the compressive stress–strain curve for wet condition; it is observed that ultimate stress for CSLW is the maximum, but strain for CSTW is the maximum among all of its peers. The observed strength may be due to the bonding of keratin elements at molecular level with its adjacent peer elements. This may have led to nearly a similar sort of results for all the cases. Due to the hydration process, it slightly reduced with results, but the dry condition values are comparable. Table 9 summarizes all distinguished and comparative case studies.

#### 4.2.4. Hardness Test

A microhardness test was carried out for four cases of big sheep horns. The observed values are depicted in Table 10. For SLD Rockwell hardness of 60, HRB is observed in comparison to 330 HV [48]. As the hardness testing machines are different, the values may not be comparable, but the results overall were better for dry conditions than for wet conditions.

#### 4.2.5. Impact Test

Impact tests were conducted in the KLE Technological University Material testing laboratory by applying Charpy and Izod conditions. The details of the observed values are provided in Table 11.

## 5. Experimental Comparative Study

**Figure 13:**
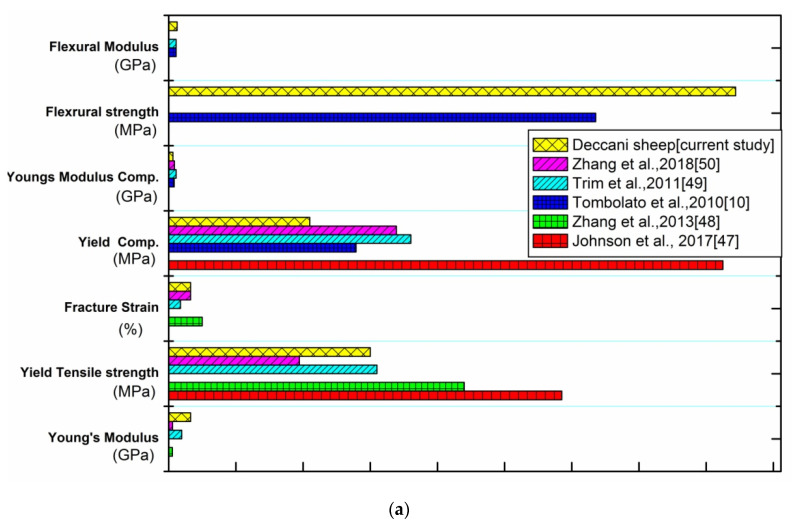

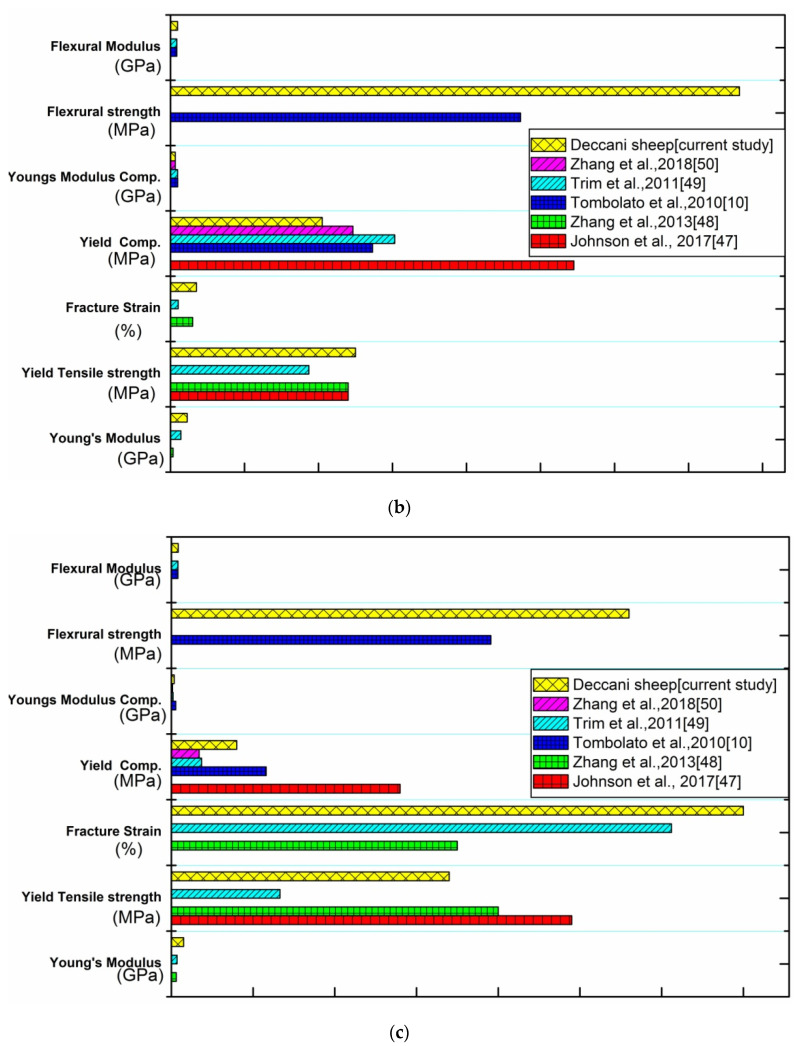

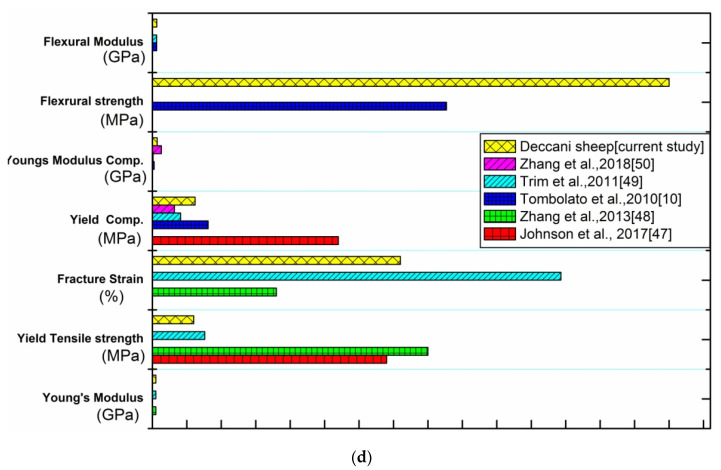
Comparison of mechanical properties with previous results at (**a**) longitudinal dry conditions (SLD), (**b**) transverse dry conditions (SLD), (**c**) longitudinal wet conditions (SLW), and (**d**) transverse wet conditions (STW).

A comparative study for any research justifies the material behavior for a mechanical and property, which is based on earlier literature noted in Figure 13. An experimental comparative study was accomplished by taking our experimental results for all four conditions (SLD, STD, SLW, STW) and comparing available results for a different breed of sheep horn at the same conditions (SLD, STD, SLW, STW), implemented by using descriptive bar charts.

In Figure 13a, where we compare mechanical properties of a different breed of sheep horn at longitudinal dry condition, we observe that big sheep horn from USA [35] has the highest tensile and compressive strength as compared with other breed sheep horn. Whereas, Young’s modulus, flexural modulus, and flexural strength of Deccani breed sheep are highest as compared with other bighorn sheep [10,35,47,48], but the failure strength of bighorn sheep from China [48] is highest among all breeds.

Figure 13b shows the comparison of mechanical properties of a different breed of sheep horn at the transverse dry condition. We observed that tensile Young’s modulus [48,49], tensile yield strength [35,47,48], and failure strain [49,50] of Deccani breed sheep horn are the highest as compared with other breed big sheep horns. Furthermore, flexural strength and flexural modulus of Deccani breed sheep horn are highest in comparison with the bighorn sheep from USA [10], whereas yield compressive strength of USA big sheep horn [35] is larger, compared to other peers.

Figure 13c shows the comparison of mechanical properties of a different breed of sheep horn at longitudinal wet conditions. It is seen that big sheep horn from USA [35] has the highest tensile and compressive strength as compared with other breed sheep horns. Young’s modulus and failure strain of Deccani breed sheep horn are highest as compared with the bighorn sheep from China and USA [48,49]. In addition, the flexural properties of the Deccani sheep horn are larger in comparison with the big sheep horn from USA [10].

Figure 13d shows the comparison of mechanical properties of a different breed of sheep horn at transverse wet conditions. We observe that the tensile yield strength of big sheep horns of China [48] is highest in comparison with other breed sheep horns. In addition, the fracture strain of big sheep horn USA [49] is larger, as compared with other peers. The flexural strength of the Deccani breed sheep horn, however, is more than the USA big sheep horn [10].

## 6. Simulation Study

Today, without simulation study, any research is incomplete and unjustified. The real-time validation of experimental work or reduction of number of experiments is feasible via simulation study [55,56,57,58]. As the virtual analysis leads to cost, material, and design optimization, further product realization and mass production becomes more compatible. A simulation study identifies potential application areas for big sheep horns with mechanical strength and stiffness properties for various analyses [59]. The details are discussed in the following sections.

### 6.1. Impact Analysis

#### 6.1.1. Geometry

**Figure 14:**
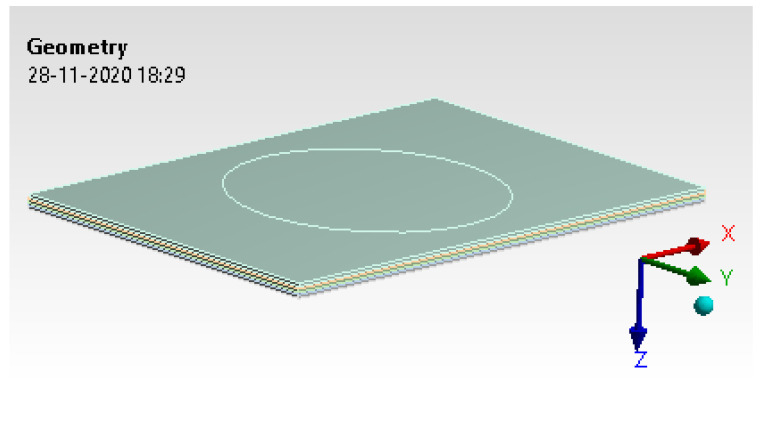
Geometry model.

The specimen was subjected to impact load testing for dimensions of 100 mm × 150 mm × 5 mm. Figure 14 depicts the pressure scoped in the highlighted surface area, which is 70 mm in diameter.

#### 6.1.2. Contact Generation

The entire coupon was subjected to holding with a fixture in the surrounding region of 70 mm dia. The layers assigned with material properties of big sheep horn are stacked one over the other with “Bonded” contact [60,61]. The analysis carried out for the stiffness method as “Pure Penalty” and the same condition maintained for further iterations [62].

#### 6.1.3. Mesh Generation

**Figure 15:**
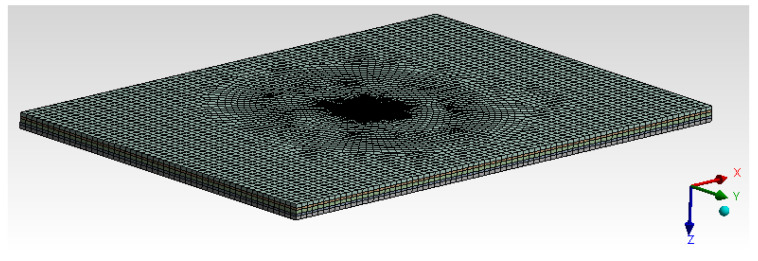
Mesh generation.

The mesh generated with Solid187 was 20-noded and hexa-dominant, with second-order elements [58,59,60] having 37,046 elements and 46,464 as nodes for the entire model, as shown in Figure 15.

#### 6.1.4. Loads and Boundary Conditions

**Figure 16:**
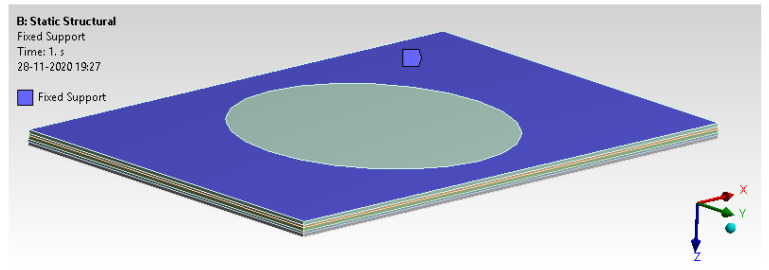
Boundary condition.

**Figure 17:**
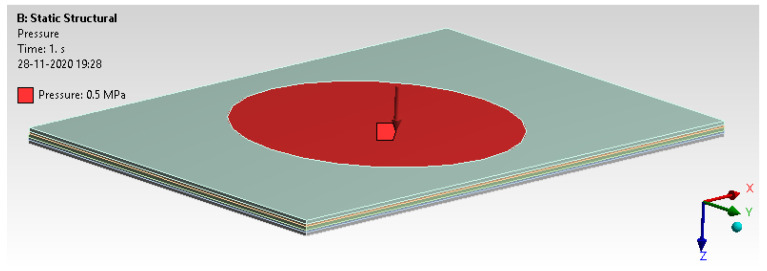
The load applied in the form of pressure.

A typical fixture arrangement mimics virtual analysis with all six degrees of freedom fixed on the top and bottom surface outside 70 mm. A pressure of 0.5 MPa is applied on the mid surface to check the possible deformation, as shown in Figure 16 and Figure 17.

**Figure 18:**
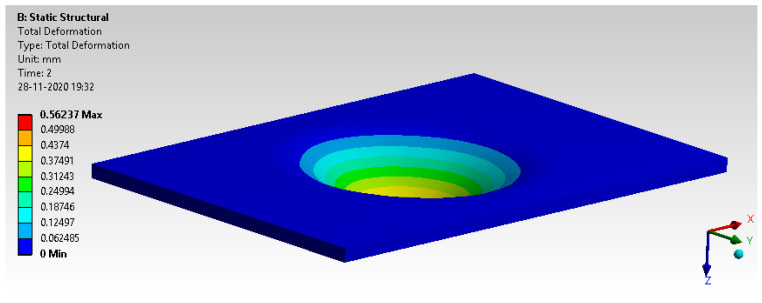
Total deformation.

**Figure 19:**
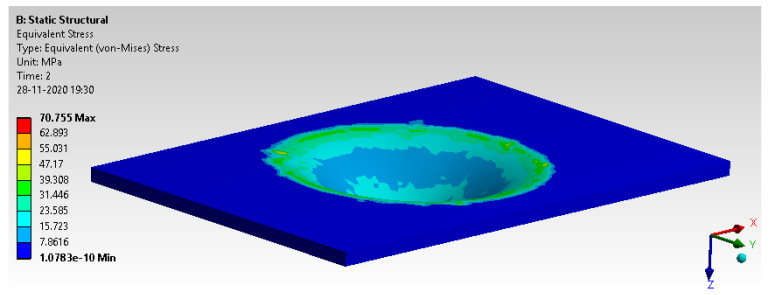
The von Mises stress.

Deformation and von Mises stress are shown in Figure 18 and Figure 19. The results observed are well within the limit of yield strength and permissible limit.

### 6.2. Fatigue Analysis

**Figure 20:**
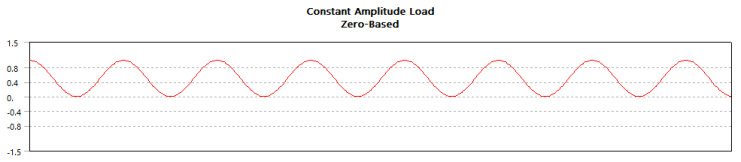
Load pattern constant.

**Figure 21:**
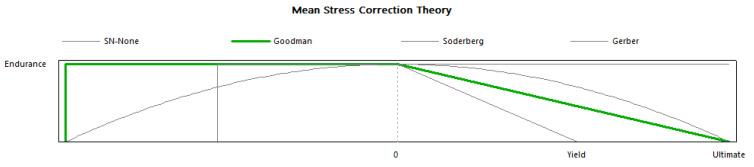
Mean stress correction theory.

When subjected to constant or fully reversible continuous load, fatigue analysis is crucial to determine the product material’s durability and life span [61,62,63]. The details of load condition and mean stress correction theory are given in Figure 20 and Figure 21.

#### 6.2.1. Life Cycle

**Figure 22:**
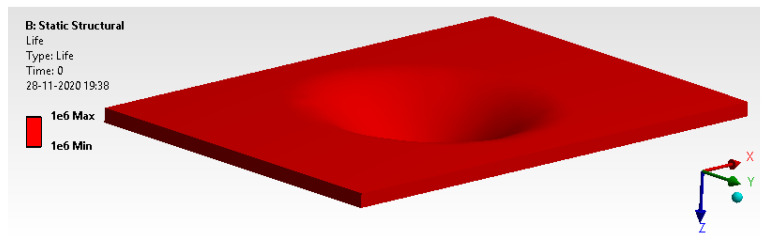
Design life cycle.

Figure 22 shows that the model has an infinite life cycle as it has over 1 × 10^5^ design cycles. Design life for the impact analysis is more than 1 lakh cycle. From Figure 22, it can be seen that life has infinite cycles.

#### 6.2.2. Damage

**Figure 23:**
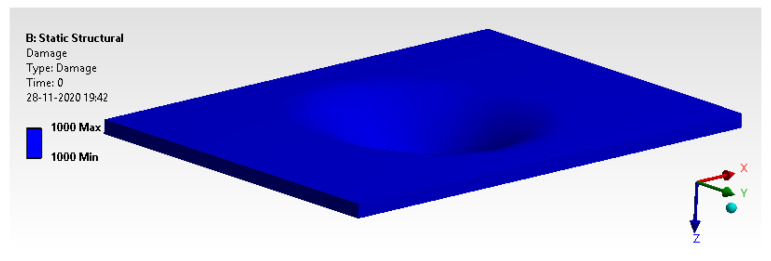
Damage.

Figure 23 illustrates that the model has a damage threshold of more than 1, which is an acceptable limit.

#### 6.2.3. Factor of Safety

**Figure 24:**
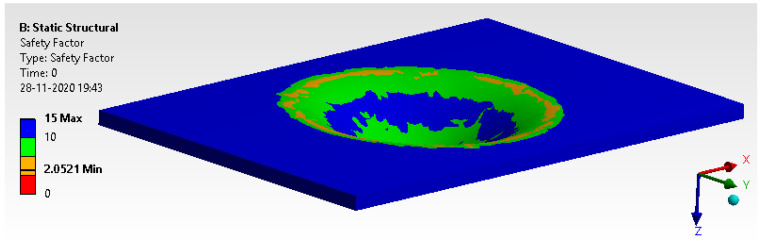
Factor of safety.

Figure 24 defines that the model has more than two as a factor of safety, which is above the threshold limit of 1.1 to 1.5. It has scope for optimization. FOS of the specimen is more than 2. This is quite obvious for the infinite cycle.

#### 6.2.4. Bi-axiality Indication

**Figure 25:**
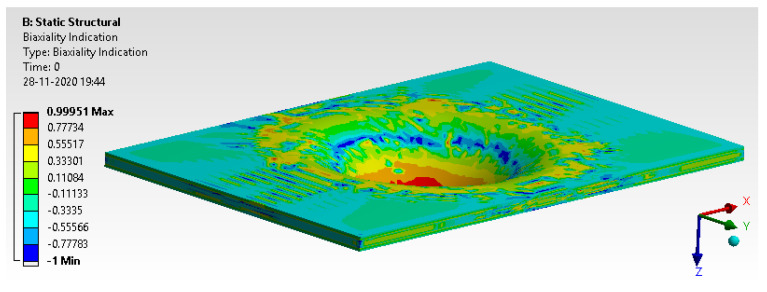
Bi-axiality indication.

Figure 25 depicts the bi-axiality indication that provides a pure compression condition. Biaxiality indication shows that the top fibers are in complete tension and bottom fibers are in compression.

### 6.3. Comparison of Experimental and Simulation Studies

**Figure 26:**
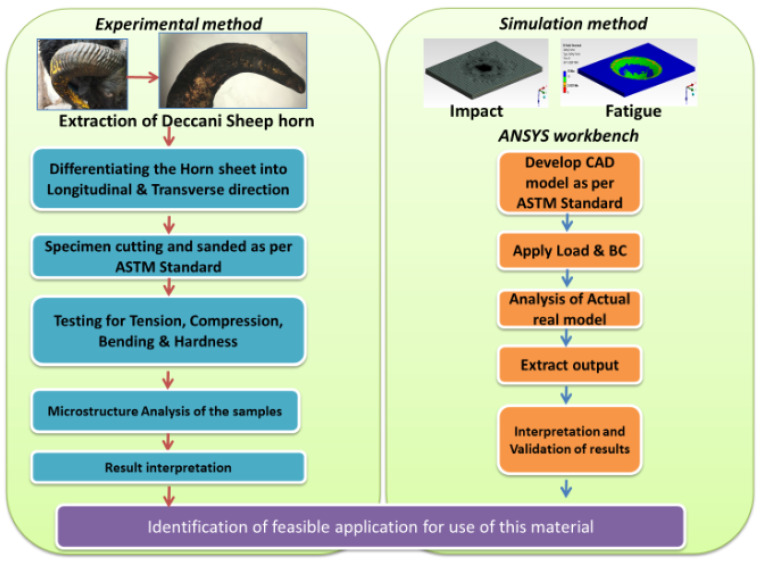
FEA and experimental comparison.

The framework with finite element analysis and experimental study comparison is carried out using the methods described in previous work [64,65,66,67,68,69] and summarized in Figure 26.

### 6.4. Applications

The exhaustive simulation study revealed the horn material is quite lucrative for application and engagement into any product as an outer layer (as it is a waste material). The novel material is tailor-made for four-wheeler vehicle bonnet, hood, skid plates, bumper, and consumer products such as mobile covers and packaging materials [70,71,72,73].

## 7. Conclusions

This exhaustive experimental work culminates in the conclusion that Karnataka’s local breed from Haveri, India, is well suited to be considered for impact-resistant applications. Further, detailed understanding provides the following conclusions:

- The density of big sheep horn keratin is 1.2 g/cc, which is the lowest among its peer breeds, and it can replace many thermo-set and thermoplastic polymers.
- Moisture absorption of SLD is 20.87%. This is by far the best among big sheep horns from other countries. For domestic sheep, it is 21%, pronghorn 38%, and mountain goat 15%. The tensile yield strength of SLD is 60 MPa, and Young’s modulus is 6.5 ± 0.5 GPa. The values are far more lucrative than many thermoplastics and fiber-reinforced polymer composites.
- The failure strain rate is 6.5 ± 0.5%, which is at par with the peer competitor species.
- Flexural strength is 168.75 MPa and the flexural modulus is 2.52 GPa. The observed values are tailor-made for use in moderate-duty load to light-duty load applications.
- Compression strength showed 43.5 MPa, which is slightly lower than its peers, but the maximum compressive stress is 563 MPa. Microhardness showed better results with 60 HRB in the case of the Rockwell hardness test.

## Figures and Tables

**Table 1:** Density test and moisture absorption properties.

Specimen	$W_{a}$ Weight of Specimen in Air (g)	$W_{w}$ Weight of Specimen in Water (g)	$\rho_{Specimen}$ the Density of Specimen (g/cc)	Moisture Absorption (%)
1	0.65	0.13	1.25	21.74
2	0.80	0.14	1.212	20

Note: The average density recorded is 1.231 g/cc.

**Table 2:** Nomenclature for coupons developed for mechanical properties.

Symbol	Designation
SLD	Stress in Longitudinal direction of Dry Specimen
SLW	Stress in Longitudinal direction of Wet Specimen
STD	Stress in Transverse direction ofDry Specimen
STW	Stress in Transverse direction of Wet Specimen

**Table 3:** Tensile properties of Deccani sheep horns.

Properties	Dry Longitudinal	Wet Longitudinal	Dry Transverse	Wet Transverse
**Young’s Modulus (GPa)**	6.5 ± 0.5	1.5 ± 0.6	4.5 ± 0.15	0.45 ± 0.5
**Yield Strength (MPa)**	53.5 ± 6.5	26 ± 6.5	43 ± 7.5	5 ± 1.5
**Ultimate Strength (MPa)**	62 ± 4.5	43 ± 3.5	46 ± 4.5	8.1 ± 2.5
**Failure Strain (%)**	6.5 ± 0.5	70 ± 0.5	5.3 ± 0.5	35 ± 0.1
**Toughness (M J/** $m^{3})$	3.14 ± 0.3	26.9 ± 0.1	1.62 ± 0.5	2.9 ± 0.1

**Table 4:** Two-way ANOVA results on the tensile properties of Deccani sheep horns-I.

Two-Way ANOVA	Yield Stress			Young’s Modulus		
	Condition (Dry and Wet)	Direction (Longitudinal and Transverse)	Interaction	Condition (Dry and Wet)	Direction (Longitudinal and Transverse)	Interaction
**DF**	1	1	1	1	1	1
**SS**	6079.5845	1221.4845	187.8845	107.973	9.072	0.5882
**MS**	6079.5845	1221.4845	187.8845	107.973	9.072	0.5882
**F- Statistic Value (df1,df2)**	746.1215 (1,16)	149.9076 (1,16)	23.0583 (1,16)	1023.126 (1,16)	85.9645 (1,16)	5.5741 (1,16)
*p*-value	7.43 × 10^−15^	1.54 × 10^−9^	0.0001956	6.66 × 10^−16^	7.78 × 10^−8^	0.03125

**Table 5:** Two-way ANOVA results on the tensile properties of Deccani sheep horns-II.

Two-Way ANOVA	Ultimate Strength			Failure Strain		
	Condition (Dry and Wet)	Direction (Longitudinal and Transverse)	Interaction	Condition (Dry and Wet)	Direction (Longitudinal and Transverse)	Interaction
**DF**	1	1	1	1	1	1
**SS**	3953.72	3120.002	618.272	10,875.9816	1507.0216	1427.881
**MS**	3953.672	3120.002	618.272	10,875.9816	1507.0216	1427.881
**F- Statistic Value (df1,df2)**	586.9466 (1,16)	463.1832 (1,16)	91.7862 (1,16)	16,824.6857 (1,16)	2331.299 (1,16)	2208.8718 (1,16)
*p*-value	$4.88\times 10^{-14}$	$3.08\times 10^{-13}$	$4.97\times 10^{-8}$	0	0	0

**Table 6:** Flexural properties of Deccani sheep horn.

Properties	Dry Longitudinal	Wet Longitudinal	Dry Transverse	Wet Transverse
Flexural Strength (M Pa)	168.75	56.25	153.75	75
Flexural Modulus (G Pa)	2.52	0.84	1.91	0.651
Ultimate Load (KN)	0.09	0.03	0.082	0.04

**Table 7:** Two-way ANOVA results on the flexural properties of Deccani sheep horns.

Two-Way ANOVA	Flexural Modulus			Flexural Strength		
	Condition (Dry and Wet)	Direction (Longitudinal and Transverse)	Interaction	Condition (Dry and Wet)	Direction (Longitudinal and Transverse)	Interaction
DF	1	1	1	1	1	1
SS	7.4164	1.0301	0.3078	42527.2531	45.7781	1500.7781
MS	7.4164	1.0301	0.3078	42527.2531	45.7781	1500.7781
F- Statistic Value (df1,df2)	118.7768 (1,16)	16.4979 (1,16)	4.929 (1,16)	3347.6965 (1,16)	4.3674 (1,16)	118.1395 (1,16)
*p*-value	8.19 × 10^−9^	0.0009064	0.0412	0	0.0456	8.51 × 10^−9^

**Table 8:** Nomenclature for coupons developed for compression testing.

Symbol	Designation
CSLD	Compressive Stress in Longitudinal direction of Dry Specimen
CSLW	Compressive Stress in Longitudinal direction of Wet Specimen
CSTD	Compressive Stress in Transverse direction of Dry Specimen
CSTW	Compressive Stress in Transverse direction of Wet Specimen

**Table 9:** Compressive properties of Deccani sheep horns.

Properties	Dry Longitudinal	Wet Longitudinal	Dry Transverse	Wet Transverse
Young’s Modulus (GPa)	1.25 ± 0.2	0.33 ± 0.1	1.01 ± 0.2	0.6 ± 0.1
Yield Strength (MPa)	41 ± 2.5	8 ± 1.2	38 ± 2.2	5.93 ± 1.2
Yield Strain (%)	4.5 ± 0.6	3.2 ± 0.2	4.2 ± 0.1	1.7 ± 0.2
Max. stress (MPa)	557.71 ± 5	557.71 ± 5	550 ± 5	520.1 ± 6

**Table 10:** Rockwell hardness test load and results for Deccani sheep horns.

Name of Hardness Test	Rockwell Hardness Test
Applied Load	60 Kgf
Indenture Used	1/16″ Ball indenture(2.5 mm)
The hardness of Deccani Sheep Horn @ base part	61 HRB/60/30
The hardness of Deccani Sheep Horn @ middle part	60 HRB/60/30
The hardness of Deccani Sheep Horn @ tip	28 HRB/60/30
60 HRB/load of 60/time of 30 s	60 HRB/60/30

**Table 11:** Impact test results for Charpy and Izod conditions for Deccani sheep horn.

Name of Test	Energy Absorbed (U) (J)	Impact Strength $\left(KJ/m^{2}\right)$	Angle of Pendulum
Izode (ASTM D256)	14	274.51	90
Charpy	20	NA	140

## Data Availability

The data presented in this study are available on request from the corresponding authors.

## References

[1] Lundrigan B. (1996). Morphology of horns and fighting behavior in the family Bovidae. J. Mammal..

[2] Kitchener A., Domenici P., Blake R.W. (2000). Fighting and the mechanical design of horns and antlers. Biomechanics in Animal Behavior.

[3] Emlen D.J. (2008). The evolution of animal weapons. Ann. Rev. Ecol. Evol. Syst..

[4] McKittrick J., Chen P.-Y., Bodde S., Yang W., Novitskaya E., Meyers M. (2012). The structure, functions and mechanical properties of Keratin. JOM.

[5] Wang B., Yang W., McKittrick J., Meyers M.A. (2016). Keratin: Structure, Mechanical properties, occurance in biological organisms, and efforts at bioinspiration. Prog. Mater. Sci..

[6] Ashby M., Gibson L., Wegst U., Olive R. (1995). The mechanical properties of natural materials. l. Material property charts. Proc. R. Soc. Land. A.

[7] Wegst U., Ashby M. (2004). The mechanical efficiency of natural materials. Philos. Mag..

[8] Meyers M.A., Chen P.-Y., Lin A.Y.-M., Seki Y. (2008). Biological materials: Structure and Mechanical properties. Prog. Mater. Sci..

[9] McKittrick J., Chen P.Y., Tombolato L., Novitskaya E.E., Trim M.W., Hirata G.A., Olevsky E.A., Horstemeyer M.F., Meyers M.A. (2010). Energy absorbent natural materials and bioinspired design stratergies: A review. Mater. Sci. Eng. C.

[10] Tombolato L., Novitskaya E.E., Chen P.Y., Sheppardd F.A., McKittrick J. (2010). Microstructure, elastic properties and deformation mechanisms of horn keratin. Acta Biomater..

[11] Kitchener A. (1987). Fracture toughness of horns and a reinterpreation of the horning behavior of bovids. J. Zool. Lond..

[12] Chen P.Y., Mckittrick J., Meyers M.A. (2012). Biological materials: Funcitonal adaptations and bioinspired designs. Prog. Mater. Sci..

[13] Makinson K.R. (1954). The elastic anisotropy of keratinous solids. The dilatational elastic constants. Aust. J. Biol. Sci..

[14] Meyers M.A., Lin A.Y.M., Seki Y., Chen P.Y., Kad B.K., Bodde S. (2006). Structural biological composites: An overview. JOM.

[15] Trim M.W., Horstemeyer M.F., Rhee H., el Kadiri H., Williams L.N., Liao J., Walters K.B., Mckittrick J., Park S.J. (2004). The effects of water and microstructure of biological materials. J. Mech. Phys. Solids.

[16] Ji B., Gao H. (2004). Mechanical properties of nanostructure of bilogical materials. J. Mech. Phys. Solids.

[17] Srivastava B., Khatri M., Singh G., Arya S.K. (2020). Microbial keratinases: An overview of biochemical characterization and its eco-friendly approach for industrial applications. J. Clean. Prod..

[18] Liu S., Xu S., Song J., Zhou J., Xu L., Li X., Zou M. (2020). Mechanical properties and failure deformation mechanisms of yak horn under quasi-static compression and dynamic impact. J. Mech. Behav. Biomed. Mater..

[19] Bonser R.H.C. (2002). Hydration sensitivity of ostrich claw keratin. J. Mater. Sci. Lett..

[20] Taylor A.M., Bonser R.H.C., Farrent J.W. (2004). The influence of hydration on the tensile and compressive properties of avian keratinous tissues. J. Mater. Sci..

[21] Li B.W., Zhao H.P., Feng X.Q., Guo W.W., Shan S.C. (2010). Experimental study on the mechanical properties of the horn sheaths from cattle. J. Exp. Biol..

[22] Feughelman M. (1997). Mechanical Properties and Structure of Alpha-Keratin Fibers: Wool, Human Hair, and Related Fibers.

[23] Bertram J.E., Gosline J.M. (1987). Functional design of horse hoof keratin: The modulation of mechanical properties through hydration effects. J. Exp. Biol..

[24] ASTM D792—20 (1991). Standard Test Methods for Density and Specific Gravity (Relative Density) of Plastics by Displacement.

[25] ASTM D5229/D5229M—20 (1992). Standard Test Method for Moisture Absorption Properties and Equilibrium Conditioning of Polymer Matrix Composite Materials.

[26] ASTM D3039/D3039M—17 (2017). Standard Test Method for Tensile Properties of Polymer Matrix Composite Materials.

[27] ASTM E9—19 (2019). Standard Test Methods of Compression Testing of Metallic Materials at Room Temperature.

[28] ASTM D790—07 (2007). Standard Test Methods for Flexural Properties of Unreinforced and Reinforced Plastics and Electrical Insulating Materials.

[29] ASTM D785—08(2015) (2015). Standard Test Method for Rockwell Hardness of Plastics and Electrical Insulating Materials.

[30] (2010). ASTM-D256-Standard-Test-Methods-for-Determining-the-Izod-Pendulum-Impact-Resistance-of-Plastics.

[31] Lin H., Mertens K., Kemps B., Govaerts T., de Ketelaere B., de Baerdemaeker J., Decuypere E., Buyse J. (2004). New approach of testing the effect of heat stress on eggshell quality: Mechanical and material properties of eggshell and membrane. Br. Poult. Sci..

[32] Bolat G., Yaman Y.T., Abaci S. (2018). Highly sensitive electrochemical assay for Bisphenol A detection based on poly (CTAB)/MWCNTs modified pencil graphite electrodes. Sens. Actuators B Chem..

[33] Vert M., Doi Y., Hellwich K.-H., Hess M., Hodge P., Kubisa P., Rinaudo M., Schué F. (2012). Terminology for biorelated polymers and applications (IUPAC Recommendations 2012). Pure Appl. Chem..

[34] Mooney B.P. (2009). The second green revolution? Production of plant-based biodegradable plastics. Biochem. J..

[35] Johnson K.L., Trim M.W., Francis D.K., Whittington W.R., Miller J.A., Bennett C.E., Horstemeyer M.F. (2017). Moisture, Anisotropy, Stress State, and Strain Rate Effects on Bighorn Sheep Horn Keratin Mechanical Properties. Acta Biomater..

[36] Hervy M., Santmarti A., Lahtinen P., Tammelin T., Lee K.Y. (2017). Sample geometry dependency on the measured tensile properties of cellulose nanopapers. Mater. Des..

[37] Fu S.Y., Lauke B., Mader E., Yue C.Y., Hu X. (2000). Tensile properties of short glass fiber and short carbon fiber reinforced polypropylene composites. Compos. A Appl. Sci. Manuf..

[38] Sabir E.C., Zervent Ünal B. (2017). The Using of Nettle Fiber in Towel Production and Investigation of the Performance Properties. J. Nat. Fibers.

[39] Bajracharya R.M., Bajwa D.S., Bajwa S.G. (2017). Mechanical properties of polylactic acid composites reinforced withcotton gin waste and flax fibers. Procedia Eng..

[40] Kandimalla R., Kalita S., Choudhury B., Devi D., Kalita D., Kalita K., Dashe S., Kotokya J. (2016). Fiber from ramie plant (Boehmeria nivea): A novel suture biomaterial. Mater. Sci. Eng. C Mater. Biol. Appl..

[41] López-Alba E., Schmeer S., Díaz F. (2018). Energy Absorption Capacity in Natural Fiber Reinforcement Composites Structures. Materials.

[42] Jia W., Gong R.H., Soutis C., Hogg P.J. (2014). Biodegradable fiber-reinforced composites composed of polylactic acid and polybutylene succinate. Plast. Rubber Compos..

[43] Vigneswaran C., Pavithra V., Gayathri V., Mythili K. (2015). Banana fiber: Scope and value added product. J. Text. Appar. Technol. Manag..

[44] Monteiro S.N. (2019). Polymer matrix composites: New fibers offer new possibilities. JOM.

[45] Mallillin A.C., Trinidad T.P., Raterta R., Dagbay K., Loyola A.S. (2008). Dietary fibre and fermentability characteristics of root crops and legumes. Br. J. Nutr..

[46] Arumugaprabu V., Uthayakumar M., Cardona F., Sultan M.T.H. (2016). Mechanical characterization of coir/palmyra waste fiber hybrid composites. IOP Conf. Ser. Mater. Sci. Eng..

[47] Zhang Q.-B., Li C., Pan Y.-T., Shan G.-H., Cao P., He J., Lin Z.-S., Ao N.-J., Huang Y.-X. (2013). Microstructure and mechanical properties of horns derived from three domestic bovines. Mater. Sci. Eng. C.

[48] Trim M.W., Horstemeyer M.F., Rhee H., El Kadiri H., Williams L.N., Liao J., Walters K.B., McKittrick J., Park S.J. (2011). The effects of water and microstructure on the mechanical properties of bighorn sheep (*Ovis canadensis*) horn keratin. Acta Biomater..

[49] Flegler S.L., Heckman J.W., Klomparens K.L. (1993). Scanning and Transmission Electron Microscopy: An Introduction.

[50] Ashby M.F., Jones D.R.H. (2012). Engineering Materials 2: An Introduction to Microstructures and Processing.

[51] Purdue University, Department of Statistics. http://www.stat.purdue.edu/~jtroisi/STAT350Spring2015/tables/FTable.pdf.

[52] Irez A.B., Zambelis G., Bayraktar E. (2019). A New design of recycled Ethylene Propylene Diene Monomer Rubber Modified Epoxy Based Composites Reinforced with Alumina Fiber; Fracture Behaviour and Damage Analyses. Materials.

[53] Warburton F.L. (1948). Determination of the elastic properties of horn keratin. J. Text. Inst..

[54] Kitchener A., Vincent J.F.V. (1987). Composite theory and effect of water on the stiffness of horn keratin. J. Mater. Sci..

[55] Yavagal P.S., Kulkarni P.A., Patil N.M., Salimath N.S., Patil A.Y., Savadi R.S., Kotturshettar B.B. (2020). Cleaner production of edible straw as replacement for thermoset plastic. Mater. Today Proc..

[56] Totla S.K., Pillai A.M., Chetan M., Warad C., Vinodkumar S.K., Patil A.Y., Kotturshettar B.B. (2020). Analysis of Helmet with Coconut Shell as the Outer Layer. Mater. Today Proc..

[57] Kohli A., Ishwar S., Charan M.J., Adarsha C.M., Patil A.Y., Kotturshettar B.B. (2020). Design and Simulation study of pineapple leaf reinforced fiberglass as an alternative material for prosthetic limb. IOP Conf. Ser. Mater. Sci. Eng..

[58] Kandekar P., Acharaya A., Chatta A., Kamat A., Patil A.Y., Kotturshettar B.B. (2020). A feasibility study of plastic as an alternative to air package in performance vehicle. IOP Conf. Ser. Mater. Sci. Eng..

[59] Patil A.Y., Banapurmath N.R., Shivangi U.S. (2020). Feasibility study of Epoxy coated Poly Lactic Acid as a sustainable replacement for River sand. J. Clean. Prod..

[60] Patil A.Y., Banapurmath N.R., Yaradoddi J.S., Kotturshettar B.B., Shettar A.S., Basavaraj G.D., Keshavamurthy R., Khan T.M.Y., Mathad S.N. (2019). Experimental and simulation studies on waste vegetable peels as bio-composite fillers for light duty applications. Arab. J. Eng. Sci..

[61] Patil A.Y., Hrishikesh N.U., Basavaraj G.D., Chalageri G.R., Kodancha K.G. (2018). Influence of Bio-degradable Natural Fiber Embedded in Polymer Matrix. Mater. Today Proc..

[62] Poornakanta H., Kadam K., Pawar D., Medar K., Makandar I., Patil A.Y., Kotturshettar B.B. (2018). Optimization of sluice gate under fatigue life subjected for forced vibration by fluid flow. J. Mech. Eng. Stroj. Gruyter..

[63] Patil V.S., Banoo F., Kurahatti R.V., Patil A.Y., Raju G.U., Afzal A., Soudagarg M.E.M., Kumarh R., Saleel C.A. (2020). A study of sound pressure level (SPL) inside the truck cabin for new acoustic materials: An experimental and FEA approach. Alex. Eng. J..

[64] Arun Y.P., Akash N., Bhavik V., Rahul K., Banapurmath N.R., Roseline M., Lekha K., Shridhar N.M. (2021). Next Generation material for dental teeth and denture base material: Limpet Teeth (LT) as an alternative reinforcement in Polymethylmethacrylate (PMMA). J. Nano Electron. Phys..

[65] Patil A.Y., Banapurmath N.R., EP S., Chitawadagi M.V., Khan T.M., Badruddin I.A., Kamangar S. (2020). Multi-Scale Study on Mechanical Property and Strength of New Green Sand (Poly Lactic Acid) as Replacement of Fine Aggregate in Concrete Mix. Symmetry.

[66] Yashasvi D.N., Badkar J., Kalburgi J., Koppalkar K., Purohit K., Patil A.Y., Fattepur G., Kotturshettar B.B. (2020). Simulation study on mechanical properties of a sustainable alternative material for electric cable cover. IOP Conf. Ser. Mater. Sci. Eng..

[67] Dhaduti S.C., Sarganachari S.G., Patil A.Y., Khan T.Y. (2020). Prediction of injection molding parameters for symmetric spur gear. J. Mol. Model..

[68] Hallad S.A., Patil A.Y., Banapurmath N.R., Hunashyal A.M., Shettar A.S., Ayachit N.H. (2017). Experimental and Numerical Validation on the Utilization of Polymer Based Nano-Composites for Structural Applications Using FEA Software Tool, Material Focus. Am. Sci. Publ..

[69] Hallad S.A., Banapurmath N.R., Patil A.Y., Hunashyal A.M., Shettar A.S. (2016). Studies on the effect of multi-walled carbon nano tube–reinforced polymer based nano-composites using finite element analysis software tool. J. Nano Eng. Nano Syst..

[70] Sharath B.N., Venkatesh C.V., Afzal A. (2021). Multi Ceramic Particles Inclusion in the Aluminium Matrix and Wear Characterization through Experimental and Response Surface-Artificial Neural Networks. Materials.

[71] Sathish T., Kaladgi A.R.R., Mohanavel V., Arul K., Afzal A., Aabid A. (2021). Experimental Investigation of the Friction Stir Weldability of AA8006 with Zirconia Particle Reinforcement and Optimized Process Parameters. Materials.

[72] Akhtar M.N., Khan M., Khan S.A., Afzal A., Subbiah R., Bakar E.A. (2021). Determination of Non-Recrystallization Temperature for Niobium Micro alloyed Steel. Materials.

[73] Nagaraja S., Nagegowda K.U., Kumar V.A., Alamri S., Afzal A., Thakur D., Kaladgi A.R., Panchal S., Saleel C.A. (2021). Influence of the Fly Ash Material Inoculants on the Tensile and Impact Characteristics of the Aluminum AA 5083/7.5SiC Composites. Materials.

